# Clinical relevance of disease‐modifying therapies for Alzheimer’s disease

**DOI:** 10.1002/alz70859_096016

**Published:** 2025-12-25

**Authors:** Alette M. Wessels

**Affiliations:** ^1^ Eli Lilly and Company, Indianapolis, IN USA

## Abstract

Current drug development efforts have led to the regulatory approval of disease‐modifying therapies for early symptomatic Alzheimer’s disease (AD).

These treatments were approved based on clinical trials that showed adequately targeting the underlying pathology of AD effectively slows the progression of cognitive and functional decline. Despite the measurable benefit on clinical outcome measures, the interpretation of these benefits has been the subject of debate.

Clinical “relevance” in AD is a latent construct that is highly multidimensional and cannot be determined by a single clinical outcome assessment or analytic measure. Meaningful treatment benefit can be demonstrated by analyzing multiple outcome measures implemented in clinical trials. An improved understanding of treatment impact on concepts that matter most to patients, clinicians, and other key stakeholders will help further translate the clinical relevance of trial findings.

This presentation will provide an overview of various approaches that confirm the clinical relevance of disease‐modifying therapies along the AD continuum.

